# Investigation of the Effect of Graphene Oxide on the Properties and Microstructure of Clay-Cement Composite Grouting Materials

**DOI:** 10.3390/ma15051623

**Published:** 2022-02-22

**Authors:** Xianzhang Ling, Xiaoyu Guo, Jing Zhong, Jinji Ma, Liang Tang, Dongliang Xing, Jianguang Su, Shengyi Cong

**Affiliations:** 1 School of Civil Engineering, Harbin Institute of Technology, Harbin 150006, China; hit_gxy@163.com (X.G.); qmy_hit@163.com (J.Z.); hitmajinji@163.com (J.M.); hit_tl@163.com (L.T.); csy_hit@163.com (S.C.); 2 Shandong Port Group Qingdao Port, Qingdao 266000, China; rongzd_hit@163.com (D.X.); mph_hit@163.com (J.S.)

**Keywords:** clay, mineral admixtures, graphene oxide, grouting, bleeding rate, rheology

## Abstract

Reductions in bleeding rates and bulk shrinkage of grouting repair materials comprise the key to solving the leakage of earth–rock dams. In this paper, an anti-seepage grouting material for earth–rock dam was developed by introducing mineral admixtures and graphene oxide (GO) nano sheets into low-cost clay–cement grouting materials and by adding polycarboxylate superplasticizers (PCs) to improve slurry viscosity. The experimental results show that the shear stress and viscosity of the slurry increase with the increase in GO concentration, and the slurry has a certain thixotropy. GO can provide a platform to promote the formation of hydration products and fill the pores of clay particles due to its high specific surface area and low volume; in this paper, the microstructure of clay–cement–graphene oxide (CCGO) grouting materials were improved. Therefore, the bleeding rate, bulk shrinkage rate, setting time and unconfined compressive strength (UCS) of the sample were macroscopically improved. In particular, the bleeding rate and bulk shrinkage rate were shown to be 0% when the content of GO reached 1.08 g/kg. Thus, the grouting anti-seepage and reinforcement performance of CCGO grouting materials were improved.

## 1. Introduction

Earth–rock dams are widely used in not only China but worldwide; they are commonly used in dam construction because of their low cost and good adaptability [1,2]. However, there are prominent safety problems related to earth–rock dams. Some earth–rock dams operate in outdated and overloaded conditions, which can result in in serious, dangerous situations during flood season. This poses a great threat to the safety of people’s lives and property. Earth–rock dams and water diversion works operate all year round. Generally, operations cannot be interrupted to carry out anti-seepage and reinforcement works; they can only be repaired online. Grouting technology can block seepage channels such as cracks and pores, enhance a crack area and stabilize a dam as a whole. Additionally, grouting technology can repair cracks and reinforce earth–rock dams online because of its low construction influence. Thus, grouting technology has become one of the most effective seepage solutions for earth–rock dams [3]. Grouting reinforcement has remarkable effects when first applied, but accidents still occur after a period of time has elapsed after grouting has been carried out. The main reason for this is that commonly used cement grouting materials have high strength, but their bleeding rates and curing shrinkage rates are high [4]. Bleeding and solidification shrinkage create pores between a stone body and soil to form seepage channels, which will lead to changes in soil particles or in the structure of a earth–rock dam, which will consequently cause seepage damage to occur in a dam body [5]. Hence, after a period of time, earth–rock dams that undergo grouting reinforcement will experience safety problems again.

Therefore, research into new grouting materials is the key to solving safety problems related to earth–rock dams. Grouting materials have been developed for a long time, since the development of pure clay slurry in the 19th century. At present, grouting materials mainly include cement, cement-water glass, clay–cement, chemical grouting materials and resins, etc. [6,7,8,9]. Among them, clay–cement grouting materials have good application value due to their low cost, good stability and strong anti-permeability [8,10]. However, the disadvantages of low strength and slow curing speed limit the application range of clay–cement grouting materials. In view of the above problem, Fei and Yi [11,12] studied the solidification effect of blast furnace slag and MgO on soil and found that the strength of blast furnace slag and MgO-solidified clay was higher than that of clay–cement specimens with the same content. The strength of the solidified sample was shown to be able to reach 1.3-4 times that of cement solidified soil when the ratio of blast furnace slag to MgO was 19:1-4:1. Cai et al. [13] studied the influence of magnesium oxide content and activity on carbonated reactive magnesia-treated silt soil and found that the greater the magnesium oxide content and activity, the better the carbonization reinforcement effect. This was shown to be true until the magnesium oxide content exceeded 27.5%, after which, the strength of the solidified soil decreased. In addition, CaCl_2_·2H_2_O, fly ash, basic oxygen furnace steel slag and other materials have been studied in an attempt to strengthen the properties of clay–cement cementitious materials. These additives were shown to participate in or promote physical and chemical interaction between cement and clay particles to produce new hydration products, so as to form a new stronger and harder matrix [14,15,16,17].

In recent years, the development of nano material technology has been perfected to ever-increasing levels. Nano material can fill the pores and provide nucleation sites of hydration products because of its very high specific surface area, surface charge and small pores. Therefore, even if these materials are added in small amounts, they can have significant impacts on the physical and chemical properties of soil [18,19]. Nano materials have increasing value in the application of clay solidification because of their excellent properties and suitable cost performance [20,21,22,23]. GO is an oxidized form of graphene, which is produced by the oxidizing graphite using a strong oxidant and then stripping it [24,25]. GO provides a platform for the growth of hydration products and promotes the formation of C-S-H gel, which makes a cementitious composite material more rigid, so as to improve the performance of a material [26,27]. Moreover, GO flakes contain functional groups such as hydroxyl, epoxide, carboxyl and carbonyl groups. These functional groups help the dispersion of GO in the cement matrix and can further improve the performance of matrix materials [28,29]. On the other hand, GO can be synthesized in large quantities from cheap, natural graphite flakes at a low cost [30]. Therefore, adding GO to clay to achieve the chemical stabilization of clay is an effective way to improve the properties of solidified clay treated with cement-based materials. Li et al. [31] proposed a method of mixing GO and cement to reinforce loess; his research determined that the mechanical properties and water resistance values were optimal when the GO content was at a level of 0.09 wt.%. In order to improve the low flexural strength and weak deformation resistance of cement-solidified expansive soil, Zhang [32] added GO to cement-expansive soil to improve the triaxial characteristics of the sample under study. The best performance was achieved when the GO content was at a level of 0.1 wt.%. Naseri [29] investigated the effect of GO flakes on the geotechnical properties of cement-soil after the ultrasonic dispersion of GO and found that the mechanical properties of cement–soil improved with the increase in GO content. SEM results showed that the pores of the soil matrix were reduced after GO was incorporated into cement–soil. In addition, Valizadeh and Zhou [19,33] found that adding a small amount of GO can significantly improve the physical and mechanical properties and microstructure of clay sand. The price of industrial GO is 2000 RMB per kg, and 0.57 kg of industrial GO is added per ton of grouting materials. The authors calculate that the average cost of GO grouting materials per ton is about 1200 RMB. In addition, the price of common pure cement grouting materials is usually about 180 RMB. Although the price of GO grouting materials is still higher than that of cement, considering the performance advantages of GO and the increasingly mature manufacturing technology of GO, GO grouting materials still have high research significance.

In summary, it can be seen that the technology of cement–soil reinforced by GO has been studied, but there are few reports that cover the application of GO-modified cement–clay grouting material. Therefore, it is necessary to systematically study the effects of different concentrations of GO on the rheological, physical and mechanical properties of cement–clay grouting materials. In this study, PCs, mineral admixtures (self-made in a laboratory) and GO with different concentrations were added to cement–clay grouting materials. The modification effect of GO on CCGO grouting materials was investigated by measuring slurry rheology, bleeding rate and UCS. Scanning electron microscopy (SEM), Energy-dispersive spectroscopy (EDS), X-ray diffraction (XRD) and Fourier transform infrared spectroscopy (FTIR) were used to study the microstructure of solidified CCGO grouting materials.

## 2. Materials and Methods

### 2.1. Materials

Oriental Portland cement (OPC), type 42.5R, produced from Harbin Swan Cement Co., Ltd. (Harbin, China) was used. The chemical composition of OPC is listed in Table 1. The clay used was Harbin silty clay. Particles with sizes less than 0.005 mm accounted for 32.7% of the mixture, particles with sizes 0.005–0.05 mm accounted for 51.9% and particle sizes greater than 0.05mm accounted for 15.4%. The liquid limit was 36.3, the plastic limit was 22.8 and the plasticity index was 13.5. The chemical composition of the clay is listed in Table 1. Mineral admixtures were self-prepared in the laboratory; they had a specific surface area of about 400 m^2^/kg and contained alkali metal aluminates, sulfates and silicates, etc. The chemical composition of the mineral admixtures is listed in Table 1. GO suspension (10 mg/mL) was supplied by Shenzhen Matterene Technology Co., Ltd. (Shenzhen, China). The diameter distribution of 8~18nm accounted for 95% of the suspension, a thickness less than 2nm accounted for 90%, and the purity reached 99.995%. PCs were procured from Tianjin Weihe Science and Technology Development Co., Ltd. (Tianjin, China) to improve the viscosity increase caused by GO.

### 2.2. Preparation of CCGO Grouting Materials

**Figure 1:**
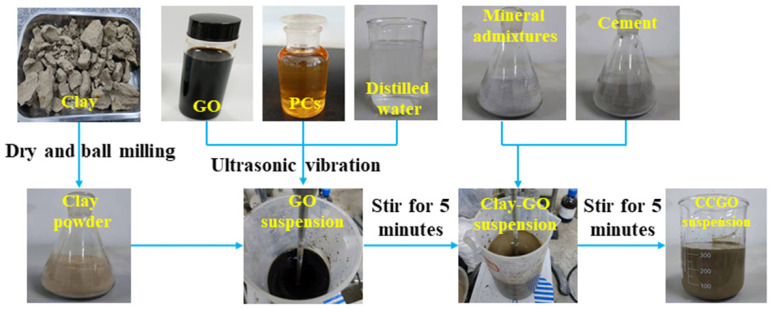
Preparation process of clay–cement–graphene oxide (CCGO) grouting materials.

The specific mix proportions of the test materials are shown in Table 2, in which supernumerary PCs and GO were added. Additionally, GO was weighed according to the mass required in Table 2 before being made into a solution, and then added into the raw material instead of water. The water:solid ratio was the ratio of the GO solution to the combined mass of all solid raw materials. The clay was dried in a drying oven at 105 °C for 1 d and cooled to room temperature and then put into a ball mill for 30 min so it was ground into a powder before the test. At the beginning of the test, the GO solution was diluted to the concentration shown in Table 2, and then, PCs were added, and the solution was vibrated for 20 min to obtain a homogeneous solution. Then, the clay was weighed and it was stirred with the GO solution for 5 min. Finally, the cement and mineral admixtures were weighed and poured into a clay–graphene suspension after it was uniformly mixed. Homogenized slurry was obtained after stirring for 5 min. The specific preparation process can be seen in Figure 1. After preparation, the slurry was poured into 40 mm × 40 mm × 40 mm molds, smoothed and placed in a curing box. The properties were tested after it was cured to the corresponding age under standard conditions [temperature (20 ± 2) °C, relative humidity 90%–95%].

### 2.3. Testing Method

An Ndj-1f continuously variable Brookfield rotational viscometer was used to test the rheology of the slurry. Rotor 21 was selected to test rheology. Test temperature is 20 °C. The shear rate increased from 5 s^−1^ to 200 s^−1^ within 10 min, and the corresponding shear stress was recorded every 10 s. The speed increased in steps, and each speed step was maintained for 30 s before recording data. Due to the pseudoplastic characteristics of the grout studied, in order to avoid the incorrect evaluation of shear stress, the modified Bingham model was adopted to accurately fit shear stress–shear rate data (Equation (1)) [34]:

(1) $$\tau =c\gamma^{2}+\eta_{p}\gamma +\tau_{0}$$

where c is a constant, τ is the shear stress, τ_0_ is yield shear stress, η_p_ is apparent viscosity and γ is shear rate.

It can be seen from the correlation coefficient in Table 3 that the modified Bingham model has high correlation and can be used to describe the rheological behavior of a slurry.

The method used to record the bleeding rate and bulk shrinkage was to pour the prepared slurry into a 250 mL measuring cylinder and start timing. Here, the volume of the slurry poured is denoted as V, the volume of bleeding water in the slurry after standing for 3 h is V_1_ (solid–liquid stratification occurred) and the solid volume is V_2_. The bleeding rate (α) and the bulk shrinkage rate (β) are given by: α = V_1_/V; β = (V − V_2_)/V.

The Initial setting time of the slurry was measured using a Vicat instrument.

An electronic universal testing machine was used to test the UCS of the samples at 3, 7 and 28 d. The samples were preserved and soaked in alcohol for subsequent XRD, FTIR and SEM tests.

The XRD patterns of samples were analyzed using an X’PERT X-ray diffractometer from Panalytical Instrument Company, Netherlands. Cu target Kα radiation with a wavelength of 0.15406 nm, tube pressure of 50 kV, a current of 100 mA, a scanning range of 5°~80° and a scanning rate of 0.1°/s was adopted.

A Thermo Nicolet 6700 spectrophotometer was used to perform FTIR in the attenuated total reflection mode (ATR). The scanning range was the mid infrared range (400–4000 cm^−1^), and the resolution was 4 cm^−1^.

A Jsm-6490lv scanning electron microscope produced by the JEPL company, Japan was used to analyze the micro morphology of the sample. Gold spraying treatment was carried out to increase the conductivity of the sample before observation, and the accelerating voltage was 15 kV. The supporting energy dispersive spectroscopic (EDS) analysis was used to analyze the relative content of point elements in the photos during SEM photography.

## 3. Results

### 3.1. Rheology

**Figure 2:**
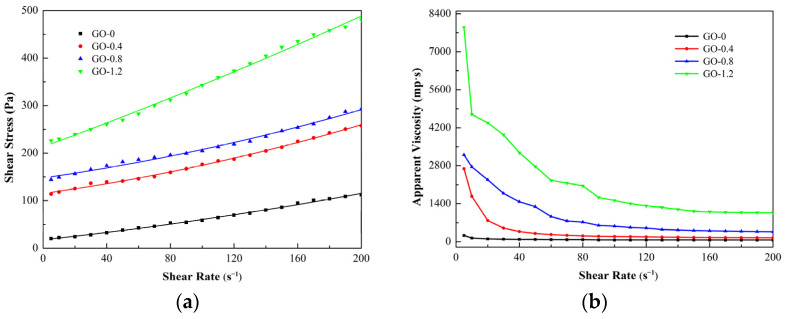
Effect of graphene oxide (GO) concentration on rheology of CCGO grouting materials: (**a**) shear rate versus shear stress; (**b**) shear rate versus viscosity.

Figure 2a,b show the rheological curve of CCGO grouting materials when the GO content is 0 g/kg, 0.36 g/kg, 0.72 g/kg and 1.08 g/kg, respectively. Figure 2a shows the effect of GO concentration on the shear rate versus shear stress of CCGO grouting materials. It can be clearly seen that with the increase in GO concentration, the shear stress of the slurry increases a great deal at the same shear rate. For example, the shear stress of slurry of GO-0 and GO-1.2 is 112.5 Pa and 481 Pa, respectively, when the shear rate is 200 s^−1^, and the shear stress increases by 3.28 times. This is mainly due to water molecules trapped in the aggregates/beams of nano materials, which is caused by the van der Waals force and electrostatic interaction between GO flakes, which then reduces the amount of free water available [35]. Moreover, the extremely high specific surface area of GO flakes adsorb a large amount of free water [29], resulting in a reduction in particle spacing and the enhancement of friction between particles in the system, which is manifested as an increase in shear stress in a macro-scopic view.

Viscosity is a hydrodynamic characteristic that hinders the relative flow between different components in a slurry, that is, the macroscopic expression of internal friction between liquid molecules, solid particles, liquid molecules and solid particles in slurry fluid. Figure 2b shows the effect of GO concentration on the shear rate versus viscosity of CCGO grouting materials. It can be seen from the figure that the apparent viscosity of all CCGO grouting materials decreased sharply with the increase in shear rate when the shear rate was small. The decrease range of apparent viscosity decreased with the continuous increase in shear rate until it reaches a plateau. This can be attributed to the fact that the flocculation structure of the slurry was destroyed and the slurry was continuously diluted under the action of shear stress; that is, the phenomenon of shear thinning occurred. This phenomenon indicates that the slurry had good thixotropic properties, and it had already been reported for other cement-based materials [36]. The apparent viscosity of the slurry increased rapidly with the increase in GO concentration, mainly because GO plays a role in connecting the flocculation structure and reduces the free water of a system, which leads to greater viscosity.

Table 3 shows the rheological parameters of CCGO grouting materials. It can be seen that the yield stress and plastic viscosity of slurry increased rapidly with the increase in GO concentration. The yield stress of cement paste increased from 17.78 Pa to 115.11 Pa, 147.86 Pa and 219.72 Pa, and the plastic viscosity increased from 0.3578 Pa·s to 0.4637 and 0.4778 and 1.2240 Pa·s when the GO content increased from 0 g/kg to 1.08 g/kg. Yield stress is the maximum limit stress to prevent plastic deformation of cement slurry, and plastic viscosity reflects the deformation speed of cement slurry [37]. Therefore, the deformation of cement paste under external forces becomes more difficult with the increase in GO concentration, which helps to form a complete structure in a seepage dam to resist water scouring.

### 3.2. Bleeding Rate and Bulk Shrinkage Rate

Bleeding rate is an important indicator of pumping stability. Only with certain pumping stability, the slurry can maintain the uniformity during the long-distance grouting pumping process to ensure that its engineering performance will not be greatly deviated during the distribution process. Thus, it can be evenly and fully filled in the cracks of the injected medium. The effect of GO concentration on bleeding rate and bulk shrinkage rate of CCGO grouting materials are shown in Table 4.

It can be seen from the data in the table that the bleeding rate of the slurry without GO was 1.27%. The bleeding rate decreased continuously with the increase in GO concentration. Additionally, the bleeding rate decreased to 0% when the GO concentration increased to 1.08 g/kg. No bleeding phenomenon was observed in the GO-1.2. Due to the water reducing and thickening effects, in which GO reduces free water in a system, the bleeding rate of the slurry was reduced. In addition, the sample used clay with good dispersion as the main raw material, which greatly improved the stability of the slurry and reduced the bleeding rate. Lastly, the aluminate incorporated by the mineral admixture improved the early hydration rate of the slurry. The ettringite generated by the reaction between aluminate and calcium sulfate contained a large amount of crystal water. The hydration process accelerated the consumption of free water and further reduced the bleeding rate of solidified grout.

Furthermore, it can be seen from Table 4 that the bulk shrinkage rate of the slurry decreased with the increase in GO concentration. The bulk shrinkage rate was 0% when the concentration of GO was 0.9 g/kg. The decrease in the bulk shrinkage rate and bleeding rate of the slurry improved the grouting filling effect and reduced the seepage channels in the formation. This avoids the problem of a reduction in the durability of a grouting-reinforced body due to the concentrated and rapid flow of groundwater in the seepage channel. Therefore, the bulk shrinkage rate and bleeding rate of slurry are important factors that affect the success of grouting. It is not difficult to see that grouting with CCGO grouting materials creates a small gap between the grouting-reinforced body and soil and does not produce a seepage channel, meaning this grouting technique will undoubtedly produce a good anti-seepage effect.

### 3.3. Setting Time

**Figure 3:**
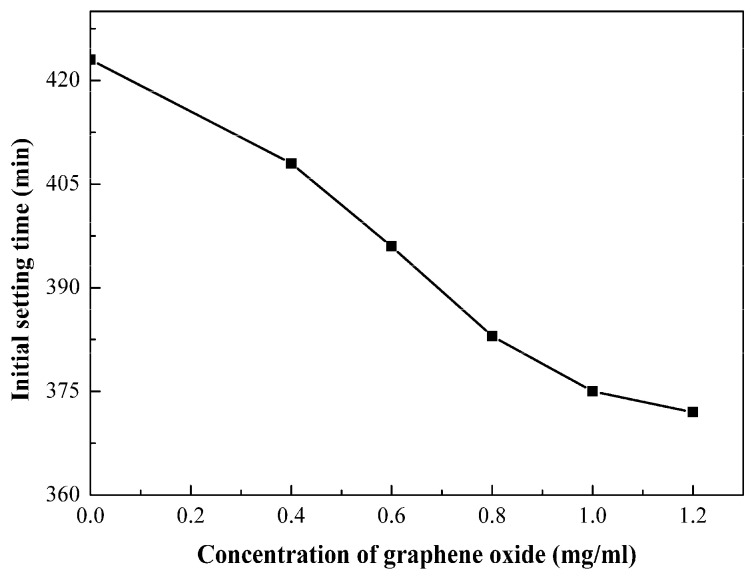
Effect of GO concentration on the initial setting time of CCGO grouting materials.

Setting time is an important parameter for grouting materials. The diffusion radius of slurry can be controlled by adjusting the setting time of slurry. Figure 3 shows the effect of GO content on the initial setting time of CCGO grouting materials. The initial setting time of slurry decreased with the increase in GO concentration. Compared with GO-0, the initial setting time of GO-0.4 increased from 408 min to 423 min. Additionally, the initial setting time was 372 min when the concentration of GO increased to 1.08 g/kg. The water reducing and hydration reaction promotion effects that GO possesses slight reduced the initial setting time. 

### 3.4. Unconfined Compressive Strength

**Figure 4:**
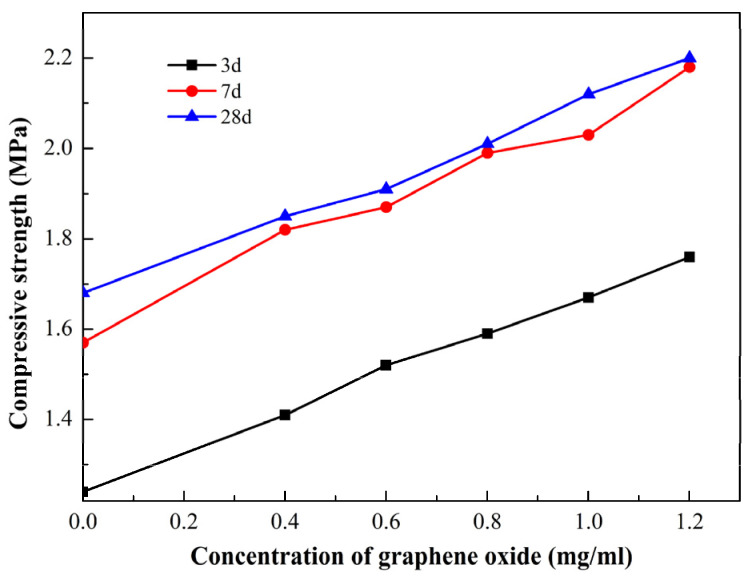
Effect of GO concentration and curing time on the unconfined compressive strength of CCGO grouting materials.

The effect of GO concentration and curing time on the UCS of CCGO grouting materials is shown in Figure 4. It can be seen from the figure that the incorporation of GO significantly increased the UCS of the stone body when the curing time was the same. The 28 d UCS of GO-0 as 1.68 MPa, and the UCS of GO-0.4 increased by 10.12% relative to GO-0, reaching 1.85 MPa. The UCS increased by 31.52% to 2.2 MPa when the GO content increased to 1.08 g/kg. In addition, the UCS of samples with the same GO concentration increased with the increase in curing age, in which the UCS underwent a large increase from 3 d to 7 d, while there was almost no increase recorded at 28 d relative to 7 d. This phenomenon can be attributed to the fact that mineral admixtures contain a large number of aluminates and sulfates, which quickly produce hydrated products such as ettringite after being added into the system, so as to improve the early strength of the sample [38]. On the whole, the sample can reach the highest strength after curing for 7 d, and the UCS of all samples is greater than 1 MPa, which is enough to meet the requirements of seepage prevention.

### 3.5. Microstructure of CCGO Grouting Materials

**Figure 5:**
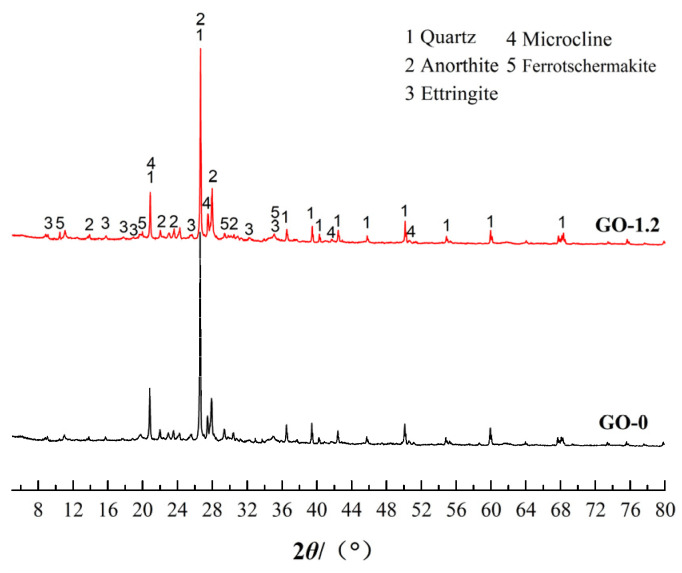
X-ray diffraction pattern of GO-0 and GO-1.2.

GO-0 and GO-1.2 were tested via XRD between 5° and 80° after 28 d of curing. The X-ray diffraction patterns of GO-0 and GO-1.2 are shown in Figure 5. The diffraction peaks of quartz, microcline, anorthite and ferrotschermakite are observed in the figure, all of which are minerals found in clay. The diffraction peak of ettringite also appears in the spectrum, which is the hydration product of the chemical reaction of the sample. The formation of ettringite is the key to improving early strength. Moreover, it can be seen that the diffraction peaks of the X-ray diffraction patterns of the two samples are not significantly different regardless of whether GO is added. The research of Balaji and Yang [39,40] shows a similar situation. This is mainly due to the fact that GO accelerates the hydration process but does not change the types of hydration products. The recognition peak of GO is not visible in the XRD diffraction pattern because of the very small amount of GO and the very high strength of the crystalline phase composition of the clay [41,42].

**Figure 6:**
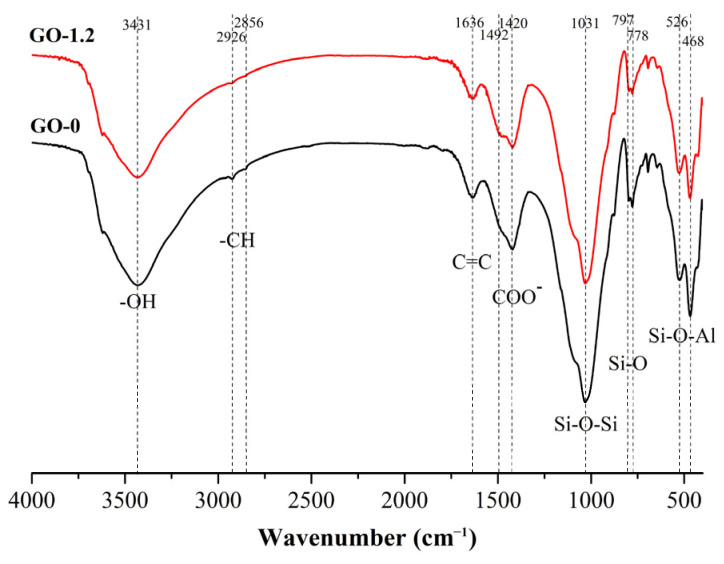
Fourier transform infrared spectroscopy spectra of GO-0 and GO-1.2.

Similar to the procedure used for the XRD test, GO-0 and GO-1.2 samples were selected to undergo FTIR testing after they were cured for 28 d. The FTIR spectra of GO-0 and GO-1.2 are shown in Figure 6. In the figure, 3431 cm^−1^ is the stretching vibration peak caused by the O-H bond of Ca(OH)_2_ [43], and 2856 cm^−1^ and 2926 cm^−1^ are the vibration absorption peaks of saturated C-H, indicating that clay contains certain organics. The stretching peak of C=C is 1636 cm^−1^, and 1420 cm^−1^ and 1492 cm^−1^ are the COO^−1^ absorption peaks. Quartz is the most difficult primary mineral to be weathered in clay. The infrared spectrum shows the existence of quartz bimodal at 778 cm^−1^ and 797 cm^−1^. The bimodal is the characteristic absorption peak of quartz [44]. The absorption peak at 526 cm^−1^ originates from the Si-O-Al bond, in which Al exists in the form of an octahedron. The absorption peak at about 468 cm^−1^ is related to the deformation and vibration of a silicon oxygen tetrahedron [45]. The results show that no new phase is formed, similar to the XRD results. All of the samples with and without GO exhibit the same peak position at the same age. Comparing the infrared spectra of GO-0 and GO-1.2, it was concluded that the presence of GO in the sample is difficult to detect by FITR. This may be due to the low amount of GO in the sample compared to the amount of all of the other components [39].

**Figure 7:**
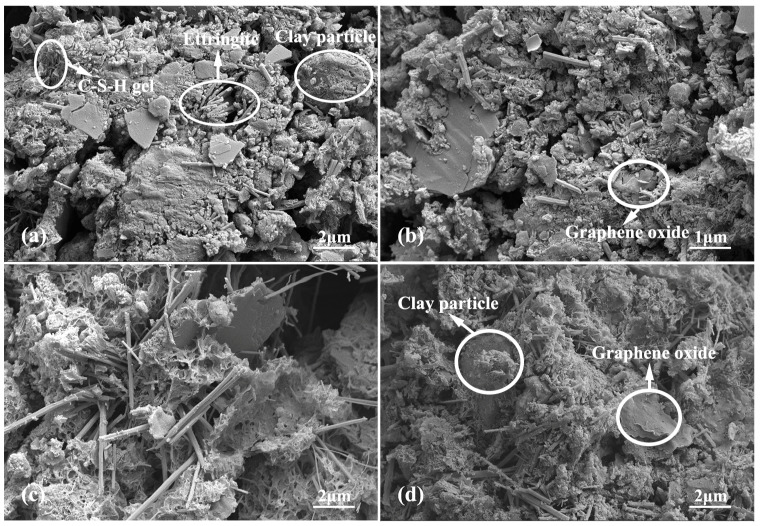
Scanning electron microscopy photos of GO-0 and GO-1.2 with curing age of 3 d and 28 d: (**a**) GO-0 with curing age of 3 d; (**b**) GO-1.2 with curing age of 3 d; (**c**) GO-0 with curing age of 28 d; (**d**) GO-1.2 with curing age of 28 d.

In order to further study the microstructure evolution of CCGO grouting materials with different curing times and GO concentrations, SEM tests were conducted on GO-0 and GO-1.2 samples with curing ages of 3 d and 28 d, respectively. Figure 7 shows the SEM photos of GO-0 and GO-1.2 with curing ages of 3 d and 28 d. According to Figure 7a, some hydration products were produced when the GO-0 without GO was cured for 3 d. The products were mainly rod-like and needle-like ettringite with faster reactions. In addition, a small amount of C-S-H gel and calcium hydroxide crystals appeared. This indicates that the alkali aluminate, sulfate and silicate of cement and mineral admixtures in the slurry began to undergo a volcanic ash reaction while they cured for 3 d. Ettringite and C-S(A)-H gel began to fill the pores of the soil and bond the soil particles, making the structure become compact. With the extension of curing age, it can be seen from Figure 7c that GO-0 generated a large amount of ettringite and C-S(A)-H cementitious hydration products after 28 d of curing. Moreover, the C-S(A)-H cementitious hydration products became the main body relative to ettringite. Ettringite embedded in the C-S(A)-H cementitious hydration products to become a skeleton, further cemented to clay particles, and the structure was more compact. The macroscopic performance shows that the UCS of the sample improved relative to the curing for 3 d.

Figure 7b,d are SEM images of the GO-1.2 sample after it was cured for 3 d and 28 d, respectively. Firstly, GO-1.2 produced a small amount of hydration products dominated by ettringite during curing for 3 d, which is similar to GO-0. Hydration products dominated by C-S(A)-H cementitious substances increased and pores became smaller in GO-1.2 cured for 28 d. In addition, in Figure 7b,d, GO flakes with the addition of GO can be seen. A large number of hydration products were attached to and around the GO flakes of GO-1.2 cured for 28 d and wrapped the interface such as glue. This structure enhanced the connection between graphene and matrix. It can also be observed that part of the ettringite was embedded in the C-S(A)-H gel, which proves that the bonding property of C-S(A)-H gel prevents the initiation and propagation of cracks. Finally, the C-S(A)-H-gelled product of GO-1.2 was shown to have a tighter structure compared with the sample without GO. Such a structure provided greater help in the improvement of the physical and mechanical properties of the sample, which is similar to the results of other studies [32]. Therefore, the UCS of the CCGO grouting materials was significantly improved, and the microstructure was denser with the incorporation of graphene oxide.

## 4. Discussion

It is obvious from the above experiments that GO causes great improvements to the properties of clay–cement grouting materials. This paper discussed the effect of the strengthening mechanism of GO on clay–cement grouting materials combined with the results of microstructure characterization. Firstly, (1) the cement was added with pure clay slurry, and the hydration reaction occurred under the action of mixing water to generate amorphous cementitious hydrate calcium aluminate hydrate, calcium silicate hydrate, calcium hydroxide and other hydration products [46]; then, (2) the active components in the mineral admixture reacted immediately to form a large amount of aluminate (Al_2_O_3_^2−^), silicate (SiO_4_^4−^) and sulfate (SO_4_^2−^) under the action of mixed water and formed crystalline hydrate ettringite with expansibility quickly, as shown in the XRD results. The formation of ettringite consumed a large amount of mixing water in the slurry and was beneficial in accelerating the progress of the cement hydration reaction. At the same time, the generated crystalline hydrate possessed a certain strength and became one of the main skeleton components of the slurry stone body. In addition, (3) exchange and absorption occurred between the low-valence cations on the surface of clay particles and the high-valence cations produced by cement hydration [8]. The electric double layer of clay became thinner with the addition of these high-valence ions, resulting in the increase in the bonding force between clay particles, so as to improve the microstructure and mechanical properties of the composites. Finally, (4) in the case of adding GO, GO was shown to be able to provide a flat platform for crystal formation and growth, because the large specific surface area of GO means it can absorb cations and form dense electron clouds. Therefore, GO has a significant nucleation effect; that is, particles may tend to concentrate on the surface of GO [19,31]. As a result, the particles are more likely to react with each other and form a dense, interwoven hydrated C-S(A)-H gel structure. As shown in Figure 7b,d, the hydration products around the GO flakes were denser. Moreover, the gap between particles was filled with the addition of GO, which improved the characteristics of the pores. Graphene oxide can be used as both a matrix and a heterogeneous nucleation catalyst to accelerate the hydration process of cement [34].

## 5. Conclusions

In this paper, mineral admixture, PCs and GO were added to clay–cement grouting material. Several tests and characterization methods were used to investigate the influence of GO concentration on the rheology, condensation time, bleeding rate, bulk shrinkage rate, UCS and microstructure of CCGO grouting materials; the following conclusions were drawn:

(1) The rheology of cement CCGO grouting materials is very sensitive to GO concentration. The shear stress of slurry increased rapidly with the increase in GO concentration. The shear stress of GO-1.2 was 3.28 times higher than that of GO-0 when the shear rate is 200 s^−1^. The apparent viscosity decreased sharply with the increase in shear rate, showing obvious thixotropy. The apparent viscosity decreased sharply with the increase in shear rate and showed obvious thixotropy;
(2) The bleeding rate, bulk shrinkage rate, initial setting time and UCS of CCGO grouting materials have a significant positive correlation with the concentration of GO, which were improved with the increase in GO concentration. The optimal GO concentration was found to be 1.2 mg/mL, where the initial setting time is 372 min, there is no bleeding rate and bulk shrinkage, and the 28 d UCS reaches 2.2 MPa.
(3) The results of XRD, FTIR and SEM show that the hydration products of CCGO grouting materials are mainly ettringite and C-S(A)-H cementitious hydration products. Moreover, SEM photos show that GO flakes can fill the pores of clay particles, which can provide a flat platform near which many hydration products grow, so that the porosity of composite materials is reduced and the structure is more compact.

## Figures and Tables

**Table 1:** Main chemical composition and content of raw materials/wt%.

Material	CaO	Al_2_O_3_	SiO_2_	MgO	SO_3_	Fe_2_O_3_	TiO_2_	K_2_O
Cement	64.39	5.61	19.69	1.25	2.80	4.14	0.31	—
Clay	2.85	17.45	64.89	1.99	—	6.53	1.12	3.46
Mineral admixture	22.64	23.14	37.23	0.65	14.65	0.43	0.04	—

**Table 2:** Ratio of CCGO grouting materials.

Number	Clay/%	Cement/%	Mineral Admixture/%	PCs (Additional)/%	GO (Additional)/g/kg	Water Solid Ratio
GO-0	81	15	4	0.3	0	0.9
GO-0.4	81	15	4	0.3	0.36	0.9
GO-0.6	81	15	4	0.3	0.54	0.9
GO-0.8	81	15	4	0.3	0.72	0.9
GO-1	81	15	4	0.3	0.9	0.9
GO-1.2	81	15	4	0.3	1.08	0.9

**Table 3:** The rheological parameters of CCGO grouting materials.

Number	Fitting Equation	τ_0_ (Pa)	η_p_ (Pa·s)	Correlation Coefficient (R^2^)
GO-0	y = 0.00065x^2^ + 0.3578x + 17.78	17.78	0.3578	0.9968
GO-0.4	y = 0.0013x^2^ + 0.4637x + 115.11	115.11	0.4637	0.9968
GO-0.8	y = 0.0012x^2^ + 0.4778x + 147.86	147.86	0.4778	0.9923
GO-1.2	y = 0.00076x^2^ + 1.2240x + 213.52	213.52	1.2240	0.9960

**Table 4:** Effect of GO concentration on bleeding rate and bulk shrinkage rate of CCGO grouting materials.

Sample Name	Bleeding Rate (%)	Bulk Shrinkage Rate (%)
GO-0	1.27	1.12
GO-0.4	0.51	0.45
GO-0.6	0.43	0.30
GO-0.8	0.31	0.15
GO-1	0.16	0
GO-1.2	0	0

## Data Availability

Data sharing not applicable.

## References

[1] Wang F., Song Z.Q., Liu Y.H., Luo B.H., Zhang W.B. (2021). Construction of the spatially varying ground motion field of a bedrock-overburden layer site and its influence on the seismic response of earth-rock dams. Arab. J. Geosci..

[2] Bayat M., Eslamian S., Shams G., Hajiannia A. (2019). The 3D analysis and estimation of transient seepage in earth dams through PLAXIS 3D software: Neural network. Environ. Earth. Sci..

[3] Park D.S., Oh J. (2017). Permeation grouting for remediation of dam cores. Eng. Geol..

[4] Güllü H., Cevik A., Al-Ezzi K., Gülsan M. (2019). On the rheology of using geopolymer for grouting: A comparative study with cement-based grout included fly ash and cold bonded fly ash. Constr. Build. Mater..

[5] Chahar B.R. (2004). Determination of length of a horizontal drain in homogeneous earth dams. J. Irrig. Drain. Eng..

[6] Azadi M.R., Taghichian A., Taheri A. (2017). Optimization of cement-based grouts using chemical additives. J. Rock. Mech. Geotech. Eng..

[7] Zhu M., Sui H., Yang H. (2018). The differences between soil grouting with cement slurry and cement-water glass slurry. IOP Conf. Ser. Earth Environ. Sci..

[8] Zhang G.J., Liu J., Li Y., Liang J.W. (2017). A pasty clay–cement grouting material for soft and loose ground under groundwater conditions. Adv. Cem. Res..

[9] Wang C.J., Liu Q., Guo C.C., Xia Y.Y. (2020). An Experimental Study on the Reinforcement of Silt with Permeable Polyurethane by Penetration Grouting. Adv. Civ. Eng..

[10] Zhang C., Yang J.S., Fu J.Y., Ou X.F., Xie Y.P., Dai Y., Lei J.S. (2019). A new clay-cement composite grouting material for tunnelling in underwater karst area. J. Cent. South. Univ..

[11] Fei J., Wang F., Al-Tabbaa A. (2016). Three-year performance of in-situ solidified/stabilised soil using novel MgO-bearing binders. Chemosphere.

[12] Yi Y.L., Liska M., Al-Tabbaa A. (2014). Properties of two model soils stabilized with different blends and contents of GGBS, MgO, lime, and PC. J. Mater. Civil. Eng..

[13] Cai G.H., Liu S.Y., Tu Y.J., Zhang D.W., Zheng X. (2015). Strength and deformation characteristics of carbonated reactive magnesia treated silt soil. J. Cent. South. Univ..

[14] Noor-ul-Amin, Muhammad K., Alam S., Gul S. (2013). Chemical activation of clay in cement mortar, using calcium chloride. Adv. Cem. Res..

[15] Jongpradist P., Jumlongrach N., Youwai S., Chucheepsakul S. (2010). Influence of Fly Ash on Unconfined Compressive Strength of Cement-Admixed Clay at High Water Content. J. Mater. Civil. Eng..

[16] Diniz D.H., Carvalho J.M.F., Mendes J.C., Peixoto R.A.F. (2017). Blast Oxygen Furnace Slag as Chemical Soil Stabilizer for Use in Roads. J. Mater. Civil. Eng..

[17] Goodarzi A.R., Akbari H.R., Salimia M. (2016). Enhanced stabilization of highly expansive clays by mixing cement and silica fume. Appl. Clay Sci..

[18] Reches Y. (2018). Nanoparticles as concrete additives: Review and perspectives. Constr. Build. Mater..

[19] Valizadeh M., Choobbasti A.J. (2020). Evaluation of nano-graphene effect on mechanical behavior of clayey sand with microstructural and self-healing approach. J. Adhes. Sci. Technol..

[20] Huang Y., Wang L. (2016). Experimental studies on nanomaterials for soil improvement: A review. Environ. Earth. Sci..

[21] Liu G., Zhang C., Zhao M.Z., Guo W.B., Luo Q. (2020). Comparison of Nanomaterials with Other Unconventional Materials Used as Additives for Soil Improvement in the Context of Sustainable Development: A Review. Nanomaterials.

[22] Figueiredo D., Correia A., Hunkeler D., Rasteiroa M. (2015). Surfactants for dispersion of carbon nanotubes applied in soil stabilization. Colloids Surf. A. Physicochem. Eng. Asp..

[23] Yao K., An D.L., Wang W., Li N., Zhou A.Z. (2019). Effect of nano-MgO on mechanical performance of cement stabilized silty clay. Mar. Georesource Geotechnol..

[24] Newell M., Garcia-Taengua E. (2019). Fresh and hardened state properties of hybrid graphene oxide/nanosilica cement composites. Constr. Build. Mater..

[25] Peng L., Xu Z., Liu Z., Wei Y.Y., Sun H.Y., Li Z., Zhao X.L., Gao C. (2015). An iron-based green approach to 1-h production of single-layer graphene oxide. Nat. Commun..

[26] Tong T., Fan Z., Liu Q., Wang S., Tan S.S., Yu Q. (2016). Investigation of the effects of graphene and graphene oxide nanoplatelets on the micro-and macro-properties of cementitious materials. Constr. Build. Mater..

[27] Wang M., Wang R.M., Yao H., Farhan S., Zheng S.R., Du C.C. (2016). Study on the three dimensional mechanism of graphene oxide nanosheets modified cement. Constr. Build. Mater..

[28] Lv S.H., Ma Y.J., Qiu C.C., Liu J.J., Zhou Q.F. (2013). Effect of graphene oxide nanosheets of microstructure and mechanical properties of cement composites. Constr. Build. Mater..

[29] Naseri F., Irani M., Dehkhodarajabi M. (2016). Effect of graphene oxide nanosheets on the geotechnical properties of cemented silty soil. Arch. Civ. Mech. Eng..

[30] Zhao L., Guo X.L., Liu Y.Y., Ge C., Chen Z.T., Guo L.P., Shu X., Liu J.P. (2018). Investigation of dispersion behavior of GO modified by different water reducing agents in cement pore solution. Carbon.

[31] Li D.B., Lei P.B., Zhang H.C., Liu J.P., Lu W. (2021). Co-Effects of Graphene Oxide and Cement on Geotechnical Properties of Loess. Adv. Mater. Sci. Eng..

[32] Zhang C., Wang W., Zhu Z.D., Li N., Pu S.Y., Wan Y., Huo W.W. (2021). Triaxial mechanical characteristics and microscopic mechanism of graphene-modified cement stabilized expansive soil. KSCE J. Civ. Eng..

[33] Zhou G.X., Zhong J., Zhang H., Hu X.Y., Wu J.L., Koratkar N., Shi X.M. (2017). Influence of releasing graphene oxide into a clayey sand: Physical and mechanical properties. RSC Adv..

[34] Chougan M., Marotta E., Lamastra F.R., Vivio F., Montesperelli G., Ianniruberto U., Bianco A. (2019). A systematic study on EN-998-2 premixed mortars modified with graphene-based materials. Constr. Build. Mater..

[35] Farooq F., Akbar A., Khushnood R.A., Muhammad W.L.B., Rehman S.K.U., Javed M.F. (2020). Experimental investigation of hybrid carbon nanotubes and graphite nanoplatelets on rheology, shrinkage, mechanical, and microstructure of SCCM. Materials.

[36] Azevedo A., Matos P.D., Marvila M., Sakata R., Silvestro L., Gleize P., Brito J.D. (2021). Rheology, Hydration, and Microstructure of Portland Cement Pastes Produced with Ground Açaí Fibers. Appl. Sci..

[37] Wang Q., Wang J., Lv C.X., Cui S.Y., Li S.Y., Wang X. (2016). Rheological behavior of fresh cement pastes with a graphene oxide additive—ScienceDirect. New Carbon. Mater..

[38] Bizzozero J., Scrivener K.L. (2015). Limestone reaction in calcium aluminate cement–calcium sulfate systems. Cement. Concr. Res..

[39] Balaji S., Swathika A. (2021). Review on mechanical and microstructural properties of cementitious composites with graphene oxide. Mater. Today Proc..

[40] Yang H.B., Monasterio M., Cui H.Z., Han N.X. (2017). Experimental study of the effects of graphene oxide on microstructure and properties of cement paste composite. Compos. Part. A Appl. Sci. Manuf..

[41] Faria P., Duarte P., Barbosa D., Ferreira I. (2017). New composite of natural hydraulic lime mortar with graphene oxide. Constr. Build. Mater..

[42] Li Q.C., He C., Zhou H., Xie Z.Y., Li D.X. (2021). Effects of polycarboxylate superplasticizer-modified graphene oxide on hydration characteristics and mechanical behavior of cement. Constr. Build. Mater..

[43] Hong Z.J., Zuo J.P., Zhang Z.S., Liu C., Liu L., Liu H.Y. (2020). Effects of nano-clay on the mechanical and microstructural properties of cement-based grouting material in sodium chloride solution. Constr. Build. Mater..

[44] Zhao S.Q., Liu G., Ou Q.H., Xu J., Ren J., Hao J.M. (2014). FTIR and ICP-MS analysis of different types of soils. Spectrosc. Spectr. Anal..

[45] Lecomte I., Liegeois M., Rulmont A., Cloots R., Maseri F. (2003). Synthesis and characterization of new inorganic polymeric composites based on kaolin or white clay and on ground-granulated blast furnace slag. J. Mater. Res..

[46] Bullard J.W., Jennings H.M., Livingston R.A., Nonat A., Scherer G.W., Schweitzer J.S., Scrivener K.L., Thomas J.J. (2011). Mechanisms of cement hydration. Cement. Concr. Res..

